# Development and Validation of Nomograms to Assess Risk Factors and Overall Survival Prediction for Lung Metastasis in Young Patients with Osteosarcoma: A SEER-Based Study

**DOI:** 10.1155/2022/8568724

**Published:** 2022-10-25

**Authors:** Zongtai Liu, Guibin Li, Haiyan Liu, Jiabo Zhu, Dalin Wang

**Affiliations:** ^1^Department of Orthopedics, Affiliated Hospital of Beihua University, Jilin, China; ^2^Department of Orthopedics, Jilin Province FAW General Hospital, Jilin, China; ^3^Department of Orthopedics, Baicheng Central Hospital, Jilin, China

## Abstract

**Background:**

To establish two nomograms to quantify the diagnostic factors of lung metastasis (LM) and their role in assessing prognosis in young patients with LM osteosarcoma.

**Methods:**

A total of 618 osteosarcoma young patients from 2010 to 2015 were included from the Surveillance, Epidemiology, and End Results (SEER) database. Another 131 patients with osteosarcoma from local hospitals were also collected as an external validation set. Patients were randomized into training sets (*n* = 434) and validation sets (*n* = 184) with a ratio of 7:3. Univariate and multivariate logistic regression analyses were used to identify the risk factor for LM and were used to construct the nomogram. Risk variables for the overall survival rate of patients with LM were evaluated by Cox regression. Another nomogram was also constructed to predict survival rates. The results were validated using bootstrap resampling and retrospective research on 131 osteosarcoma young patients from 2010 to 2019 at three local hospitals.

**Results:**

There were 114 (18.45%) patients diagnosed as LM at initial diagnosis. The multivariate logistic regression analysis suggested that T stage, N stage, and bone metastasis were independent risk factors for LM in newly diagnosed young osteosarcoma patients (*P* < 0.001). The ROC analysis revealed that area under the curve (AUC) values were 0.751, 0.821, and 0.735 in the training set, internal validation set, and external validation set, respectively, indicating good predictive discrimination. The multivariate Cox proportional hazard regression analysis suggested that age, surgery, chemotherapy, primary site, and bone metastasis were prognostic factors for young osteosarcoma patients with LM. The time-dependent ROC curves showed that the AUCs for predicting 1-year, 2-year, and 3-year survival rates were 0.817, 0.792, and 0.815 in the training set and 0.772, 0.807, and 0.804 in the internal validation set, respectively. As for the external validation set, the AUCs for predicting 1-year, 2-year, and 3-year survival rates were 0.787, 0.818, and 0.717.

**Conclusions:**

The nomograms can help clinicians strengthen their personal decision-making and can improve the prognosis of osteosarcoma patients.

## 1. Background

Osteosarcoma is the most frequent primary malignant bone tumor among people [1]. Osteosarcoma originates from primitive mesenchymal cells and occurs mostly in bone and rarely from tissue [2]. The incidence of osteosarcoma is around one to three cases annually per million individuals worldwide, and the highest incidence is in children and young adults [3]. The lung is the most common site of metastasis in patients with osteosarcoma, and it is the main cause of death in osteosarcoma patients [4, 5]. Studies have shown that the prognosis of patients with lung metastasis is very poor, with a 5-year survival rate of about 25% [6]. According to previous studies, about 15–20% of patients have visual evidence of metastasis and almost 90% of the metastasis occurs in the lung at the time of diagnosis, while the remaining 80–90% of patients may have micrometastasis that are still subclinical or undetectable [5, 7, 8]. Furthermore, even 30–40% of patients with localized tumors would have a local or distant recurrence in the first 2–3 years, and approximately, 90% of relapses are LM [9, 10]. Therefore, predicting the occurrence of LM in osteosarcoma and providing more personalized treatment advice for patients with LM have important clinical significance for improving prognosis.

Although systematic treatment was developed after 1970, polychemotherapy and surgery still remain insufficient. In the last 50 years, the survival rates of osteosarcoma patients have not improved significantly, regardless of whether metastasis occurred [11]. The Musculoskeletal Tumor Society and the American Joint Committee on Cancer are the most widely used staging system. However, these systems are still limited in predicting the occurrence of LM and in guiding the prognosis of patients with LM. The nomogram has been widely used in predicting the prognosis of cancer patients as a tool that can combine different kinds of variables [12]. By combing important variables, the nomogram can individually estimate the probability of events such as the overall survival rate more accurately than the traditional staging systems [12–14]. In addition, the development of data science makes it possible to use big data from medical databases for statistical analysis. Data mining technology has become the frontier of medical research due to its good performance in assessing patient risk and helping to build clinical decision-making of disease prediction models [15, 16]. Considering that there are no studies focused on establishing predictive models for the diagnosis and prognosis of LM in osteosarcoma, especially for children and youth patients, the aim of the present study was to develop two nomograms to predict the probability of LM and the survival rates of young osteosarcoma patients, respectively.

## 2. Methods

### 2.1. People Selection

The data contained in this retrospective study were downloaded from the SEER database (version 8.3.6). Patients with a diagnosis of osteosarcoma between 2010 and 2015 were included in this study. Patients were randomized into training sets and internal validation sets with a ratio of 7:3. In addition, we retrospectively collected data for young osteosarcoma patients from three local hospitals as an external validation set. The training sets were used to establish the nomograms, while the validation sets were used to validate the established nomograms.

The inclusion criteria were as follows: (1) osteosarcoma was the primary tumor; (2) the age at diagnosis was 24 or less; (3) patients were histologically confirmed; (4) patients with complete clinicopathological features, demographic information, and follow-up information. Exclusion criteria were as follows: (1) patients who were diagnosed with the state of death; (2) the aforementioned information was missing; (3) patients with survival time <1 month.

Analysis of anonymous data from the SEER database is exempt from medical ethics review and does not need informed consent. The content of this retrospective research did not involve human subjects or personal privacy. Hence, informed consent from patients was not required in this study.

### 2.2. Study Variables

We selected 8 variables based on the patient-specific information from the SEER database for the study of risk factors of LM in osteosarcoma, including age, sex, race, tumor size, primary site, T stage, N stage, and bone metastasis. Patients' ages were divided into three stages: 0–8 years, 9–16 years, and 17–24 years. Tumor size was classified into three grades, ranging from 0 to 100 mm, 101 to 200 mm, and greater than 200 mm. Subsequently, the information on grade, histological type, surgery, radiotherapy, and chemotherapy were included in the analysis of prognostic factors of young osteosarcoma patients with LM. In the present study, the overall survival rate was defined as the survival time from diagnosis to death from any cause.

### 2.3. Construction and Validation of Nomograms

To start with, the total patients included in this study were enrolled in the first cohort to study the risk factors of LM in osteosarcoma. After that, those diagnosed with LM among the total patients were further set up as a second cohort for survival analysis. Patients in each cohort were randomized into training and internal validation sets with a ratio of 7:3. Data from local hospitals were used as external validation sets. The training sets were used to establish the nomograms, while the validation sets were used to validate the established nomograms.

In the study of risk factors for LM, univariate and multivariate logistic regression analyses were applied and the risk factors were chosen to construct a nomogram. The univariate and multivariate Cox proportional hazard regression analyses were performed to identify the independent prognostic factors in the survival analysis, and a prognostic nomogram was constructed based on the prognostic factors. Receiver operating characteristic (ROC) curves or time-dependent ROC curves for each nomogram were established, and the corresponding area under the curve (AUC) was used to evaluate the discrimination of nomograms. Furthermore, the calibration curves and decision curve analysis (DCA) for each nomogram were also established to estimate the clinical application value. Finally, to further verify the value of the prognostic nomogram, we divided the patients into two risk levels according to the cut-off values of total nomogram points. The Kaplan–Meier (K-M) survival curves with a log-rank test were generated, and we established the scatter diagram to make it more visual.

### 2.4. Statistical Analysis

This study adopted SPSS 25.0, X-tile (version 3.6.1), and *R* software (version 4.0.1) for all statistical analyses. Age and tumor size were classified into categorical variables and expressed as frequency (proportions). The chi-square test and the rank-sum test were used for categorical data. *R* packages including “rms” and “regplot” in *R* software were employed to draw graphics. All *P* values were two‐sided, and *P* values <0.05 were considered statistically significant.

## 3. Results

### 3.1. The Characteristics of the Population

In the present study, a total of 618 young osteosarcoma patients from the SEER database were included. Among them, 114 (18.45%) patients were diagnosed as LM at initial diagnosis. Furthermore, 434 (70.00%) and 184 (30.00%) patients were randomly divided into the training set and the internal validation set in the first cohort. In addition, data of 131 patients from our local hospitals were collected as an external validation set. There were no significant differences between the training and two validation sets, except for race and LM (*P* > 0.05) (Table 1).

### 3.2. Risk Factors of LM in Osteosarcoma Patients

To identify the risk factors of LM in young osteosarcoma patients, a univariate logistic analysis was first performed. The results indicated that four variables were related to LM in young osteosarcoma patients, including T stage (T2: OR = 4.166, 95% CI: 2.283–7.603, *P* < 0.001; T3: OR = 11.067, 95% CI: 2.876–42.583, *P* < 0.001; TX: OR = 38.733, 95% CI: 7.380–203.290, *P* < 0.001), N stage (N1: OR = 26.106, 95% CI: 5.663–120.346, *P* < 0.001; NX: OR = 10.680, 95% CI: 3.200–35.645, *P* < 0.001), tumor size (101–200: OR = 2.005, 95% CI: 1.183–3.401, *P* = 0.010; >200: OR = 6.406, 95% CI: 3.221–12.742, *P* < 0.001), and bone metastasis (OR = 18.889, 95% CI: 5.256–67.887, *P* < 0.001). The abovementioned variables were further included in the multivariate logistic regression analysis subsequently. The results showed that N stage (N1: OR = 15.855, 95% CI: 2.932–85.742, *P* = 0.001; NX: OR = 9.123, 95% CI: 2.440–34.107, *P* = 0.001), T stage (T2: OR = 3.871, 95% CI: 2.052–7.300, *P* < 0.001; T3: OR = 4.739, 95% CI: 0.876–25.653, *P* = 0.071; TX: OR = 18.915, 95%CI: 3.023–118.342, *P* = 0.002), and bone metastasis (OR = 13.966, 95%CI: 3.423–56.976, *P* < 0.001) were independent risk factors for LM in newly diagnosed young osteosarcoma patients (Table 2).

### 3.3. Development and Validation of the Nomogram for Prediction of LM

**Figure 1:**
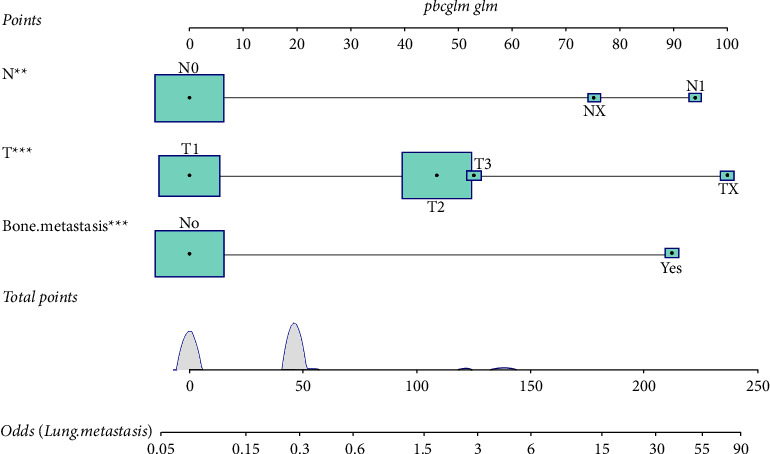
The nomogram incorporating three clinical variables for predicting the risk of LM in young osteosarcoma patients.

**Figure 2:**
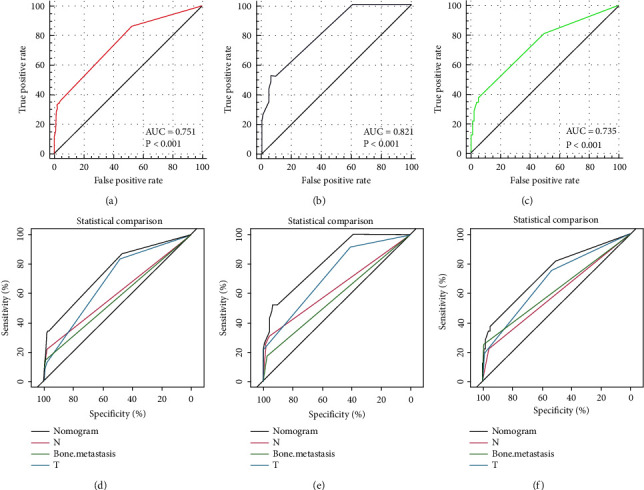
The receiver operating characteristic curve of the nomogram in the training set (a), internal validation set (b), and external validation set (c). Comparison of the values of the area under the curve between the nomogram and single independent risk factors in the training set (d), internal validation set (e), and external validation set (f).

**Figure 3:**
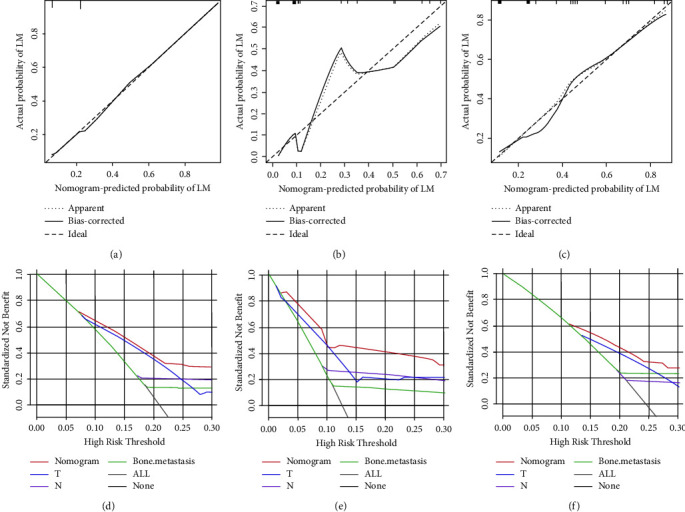
The calibration curve of the nomogram in the training set (a), internal validation set (b), and external validation set (c); the decision curve analysis of the nomogram in the training set (d), internal validation set (e), and external validation set (f).

A nomogram was established based on the results of multivariable logistics regression (Figure 1). The ROC curves of each set were constructed, and the corresponding AUC values were 0.751, 0.821, and 0.735 in the training set, internal validation set, and external validation set, respectively (Figures 2(a)–2(c)). Furthermore, ROC curves were constructed for each independent factor. The result suggested that the nomogram had a significant advantage in the accuracy of prediction compared with other variables. As shown in Figures 2(d)–2(f), the AUC of the nomogram was higher than that of other independent risk factors, both in the training set and the validation set. In the diagnostic model, the TN stage was a risk factor and the DCA curve was directly compared with it, but the TN stage was not a risk factor in the prognostic model, so the comparison was not made. Therefore, we further compared modeling with our model using TN staging (Supplementary Figure 1, 2). In the internal validation set, one-year DCA images could not be shown due to too few samples and too low TN staging decision income. In other images, it can be seen that our model has a better prediction effect. The calibration curves of each set showed a robust calibration of the nomogram (Figures 3(a)–3(c)), and the DCA curves of each set indicated that the nomogram had higher net benefits than any other independent risk factors (Figures 3(d)–3(f)).

### 3.4. Survival Analysis for Patients with LM

A total of 114 young osteosarcoma patients with LM from the SEER database and 32 patients from local hospitals were included for the survival analysis. The baselines of these patients were summarized in Table 3. The univariate Cox proportional hazard regression analysis showed that age, race, primary site, N stage, surgery, chemotherapy, and bone metastasis were independent prognostic factors. The multivariate Cox proportional hazard regression analysis suggested that age (9–16: HR = 0.264, 95% CI: 0.081–0.861, *P*=0.027; 17–25: HR = 0.621, 95% CI: 0.196–1.971, *P*=0.419), primary site (other: HR = 6.866, 95% CI: 1.538–30.655, *P*=0.012; spine/pelvis: HR = 2.126, 95% CI: 0.664–6.810, *P*=0.204; upper limb: HR = 2.138, 95% CI: 0.998–4.580, *P*=0.051), surgery (HR = 0.400, 95% CI: 0.188–0.855, *P*=0.018), chemotherapy (HR = 0.123, 95% CI: 0.037–0.411, *P*=0.001), and bone metastasis (HR = 3.981, 95% CI: 1.695–9.350, *P*=0.002) were prognostic factors for young osteosarcoma patients with LM (Table 4).

#### 3.4.1. Development and Validation of the Prognostic Nomogram for Assessing Survival

**Figure 4:**
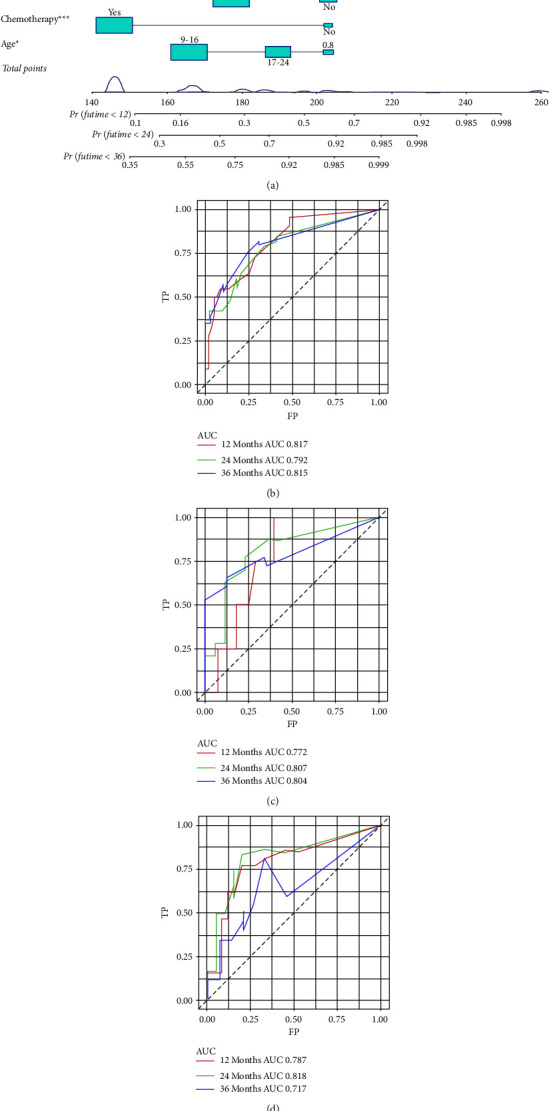
Prognostic nomogram for predicting the overall survival of young osteosarcoma patients with LM (a); three time-dependent receiver characteristic curves to show the discrimination of prognostic nomogram in the training set (b), internal validation set (c), and external validation set (d).

**Figure 5:**
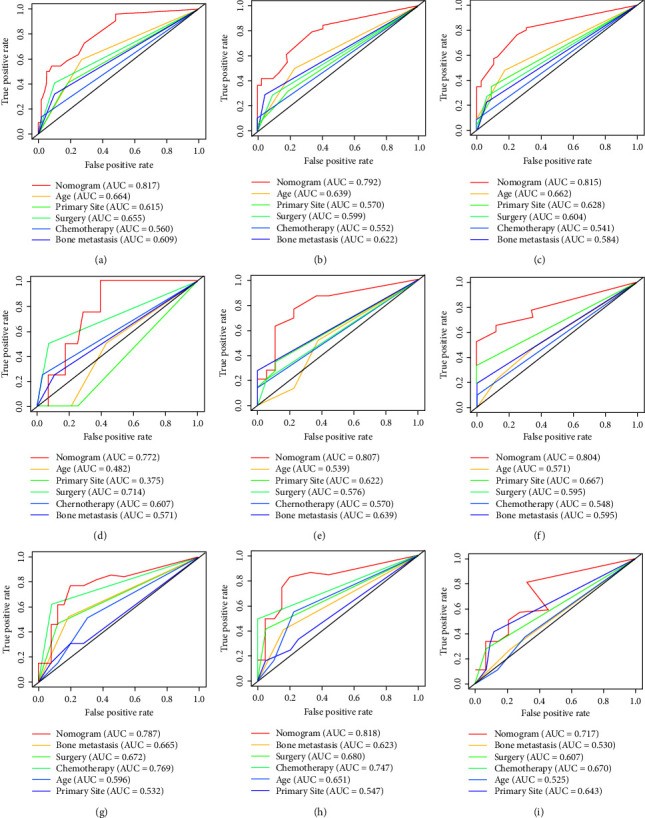
Comparison of the values of the area under the curve between the nomogram and single independent factors. 12-month, 24-month, and 36-month survival in the training set (a-c), internal validation set (d-f), and external validation set (g-i).

**Figure 6:**
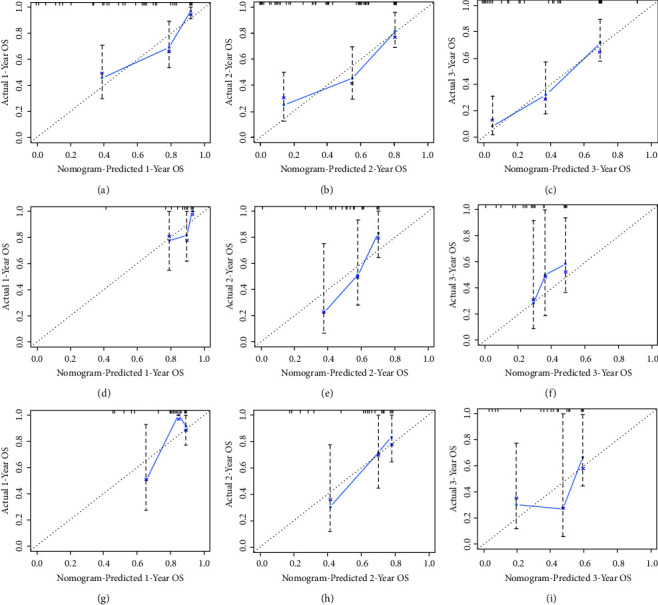
Calibration curves of 12-month, 24-month, and 36-month survival rates in the training set (a-c), internal validation set (d-f), and external validation set (g-i).

**Figure 7:**
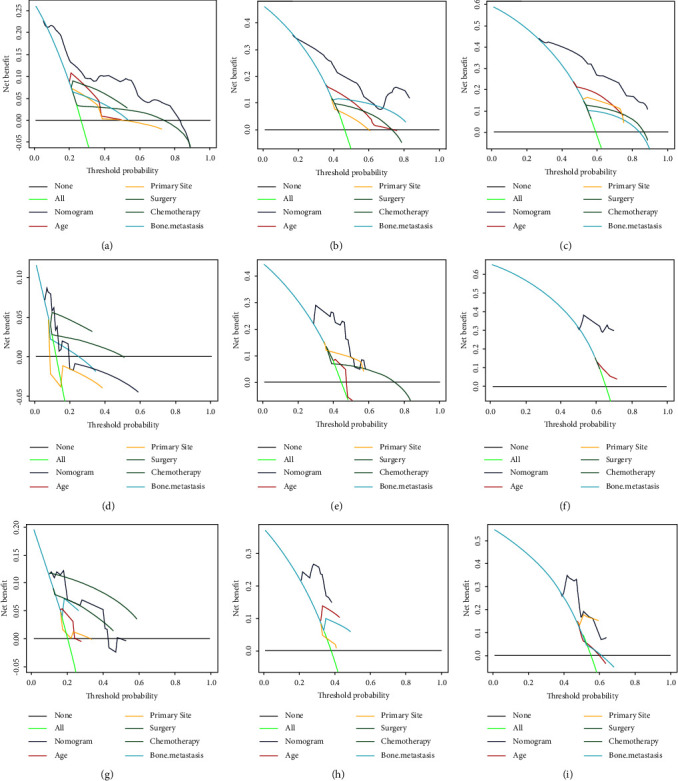
Comparison of decision curve analysis between the prognostic nomogram and single independent factors. 12-month, 24-month, and 36-month survival rates in the training set (a-c), internal validation set (d-f), and external validation set (g-i).

**Figure 8:**
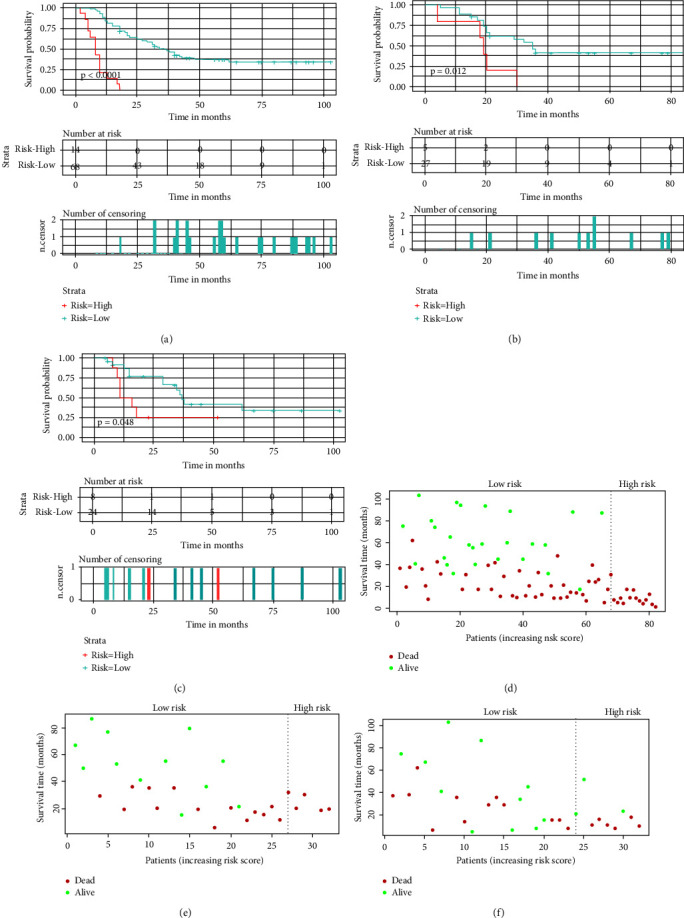
Risk stratification for young osteosarcoma patients with LM. The survival curve of two risk groups in the training set (a), internal validation set (b), and external validation set (c); a scatter diagram to show the survival status of patients in the training set (d), internal validation set (e), and external validation set (f).

Based on the Cox regression analysis, a prognostic nomogram was constructed (Figure 4(a)). The time-dependent ROC curves showed that the AUC for predicting 1-year, 2-year, and 3-year survival rates were 0.817, 0.792, and 0.815 in the training set; 0.772, 0.807, and 0.804 in the internal validation set; 0.787, 0.818, and 0.717 in the external validation set, respectively (Figures 4(b)–4(d)). The time-dependent ROC curve comparisons of the prognostic nomogram and other factors were also constructed both in the training set and two validation sets. The AUC of the nomogram was higher than that of age, primary site, surgery, chemotherapy, and bone metastasis at 1, 2, and 3 years (Figures 5(a)−5(i)). In addition, the calibration curves indicated a good consistency between the nomogram-predicted overall survival rate and the actual overall survival rate at 1, 2, and 3 years in each set (Figures 6(a)−6(i)). The DCA was used to evaluate the clinical utility, and each set had more decent performance than single independent prognostic factors (Figures 7(a)−7(i)). Furthermore, patients were divided into two risk groups according to the cut-off points. The optimal cut-off point for the total score was determined by X-tile software. A score of less than 196 was considered the low-risk group, and more than 196 was considered the high-risk group. The K-M survival curves for each set were generated. It was suggested that the patients in the high-risk group have a worse prognosis (Figures 8(a)−8(c)) than those in the low-risk group. Eventually, three scatter diagrams were also generated to show the difference between different risk groups. With the increase in the risk score, the survival rate of patients declines, and the survival time also decreases (Figures 8(d)–8(f)).

## 4. Discussion

Despite being the most common primary malignant bone tumor in children and young adults, osteosarcoma can still be considered a very rare disease. Approximately, 400 new cases are diagnosed annually in children and young adults in the USA [17]. Cohort studies of patients with osteosarcoma have been difficult due to the scarcity of patients and the high heterogeneity at the genetic level [18]. Considering that older patients with osteosarcoma may have great heterogeneity with younger patients, this study only focused on children and young adults. In the present study, we reviewed and analyzed previous valuable data from the SEER database, and the results showed that 114/618 (18.45%) of young patients had LM at the time of diagnosis. Although this percentage is slightly higher than the value reported in the previous studies, this may be due to the development and popularity of clearer tests [19].

In the study of the diagnostic factor of LM, T stage, bone metastasis, and N stage were identified as the most meaningful factors. T stage as an independent risk factor for predicting distant metastasis has been reported in previous studies [13, 14]. Bone metastasis and N stage were also independent risk factors. Tumor metastasis requires the support of the corresponding microenvironment, and these factors may indicate that the microenvironment is out of order and suitable for osteosarcoma metastasis [20–22]. Wang et al. [23] reported that the monocyte ratio and neutrophil/lymphocyte ratio could predict metastasis in osteosarcoma patients. Comparing the diagnostic model with single factors by ROC curves and DCA curves, the nomogram had better predictive performances. There have been some previous studies on the modeling of LM in osteosarcoma [13, 14, 24]. However, none of these models have been validated by local data, which may make it difficult to adapt the models' conclusions to other patients. In addition, the patient cohorts in these studies included older patients, which may make the conclusion less conclusive. Patients over 60 are often associated with Paget's disease and probably represent a distinct biological process [6]. To our knowledge, this is the first multivariate model to predict the risk of LM in young osteosarcoma patients, which shows good prediction performance in a variety of validation methods.

Osteosarcoma patients with LM or relapse have an unquestionably worse prognosis, with a 1-year survival rate of only 60%, compared with nearly 90% for localized tumors. The 2-year and 3-year survival rates of osteosarcoma with LM were less than 50%, and the gap between the survival rates of LM and localized osteosarcoma was further widened [17]. Today, the treatment of osteosarcoma is not limited to a single method, but multidisciplinary treatment has made a significant contribution to improving the survival time and quality of patients [5, 6]. Since 1970, almost all patients with osteosarcoma have been recommended to receive neoadjuvant chemotherapy or chemotherapy. At present, doxorubicin, cisplatin, high-dose methotrexate, and ifosfamide are considered the most active agents against osteosarcoma, but the ideal combination remains to be defined [25–28]. It is worth noting that the side effects of chemotherapy drugs may also have an adverse effect on the prognosis of patients. Serotonin antagonists alone or in combination with dexamethasone have been widely used to reduce chemotherapy-induced emesis [29, 30]. Lewis et al. reported that the use of G-CSF can help increase the dose of treatment and improve histological response [31]. The surgical removal of all evident diseases has been identified to be effective in improving the prognosis of patients with osteosarcoma. A cohort study involving 202 patients with metastasis reported that surgery could significantly improve prognosis. In addition, patients with unresectable macroscopic tumors had a five-fold higher risk of dying than patients who underwent a complete surgical resection of all tumor deposits [32]. More advanced biomedical engineering combined with preoperative chemotherapy has gradually transformed traditional amputation into limb salvage surgery. However, the surgery of axial bone remains particularly challenging because of the higher risk of recurrence and the common complications associated with reconstruction [33, 34]. A careful exploration must be made for potential microscopic metastasis. Due to the limited imaging resolution, CT scans may underestimate the number of LM. Pastorino et al. reported that bilateral exploration by open thoracotomy would find 16% more microscopic modules than CT, and these nodules were mostly metastatic tumors after postoperative pathological examination [35]. As a minimally invasive technique popularized in recent years, video-assisted thoracoscopy can significantly shorten the length of hospital stay and reduce the perioperative risk [36]. However, thoracoscopy also faces the problem of target miss by the limited intraoperative field of vision and faces difficulty in removing the deep nodules due to the limited equipment. Therefore, the choice of surgery requires a detailed preoperative assessment. With the discovery of more biomarkers related to osteosarcoma, immunotherapy and targeted therapy provide a new direction for the treatment of osteosarcoma, but the data on these new methods have not been encouraging [4, 37]. Osteosarcoma was long considered a radioresistant tumor. However, a study suggested that radiotherapy may be useful for patients who are unable to accept complete resection or have microscopic residual tumor nodules [33]. In recent years, several new radiotherapy techniques make it possible to simultaneously maintain a high radiation dose of the lesion and protect other important organs [38, 39]. Unfortunately, radiotherapy was not an independent risk factor in our study of prognosis factors in patients with LM. Age and more complex metastatic states, such as combining bone metastasis, have also been suggested to be associated with prognosis in patients with osteosarcoma [8, 32, 40, 41]. The age group of 0–8 years had the highest risk of death, which may be due to poorer immune function and less tolerance to treatment such as surgery compared with other age groups. The risk was lower in the 9–16-year group than in the 17–24-year group, which may be due to the better physical repair ability of this group. In addition, since the incidence was highest in the 9–16-year group, this may lead to more timely examination and mature treatment. Compared to similar previous studies, the proposed model had a better predictive performance because it was more targeted [13, 42]. In addition, we used local patient data for external validation, which means that our model had better external adaptability. Similar to the diagnostic model, we further compared the prognostic model with single risk factors by ROC curves and DCA curves. The AUCs of the nomogram were higher than all the corresponding single factors. In the comparison of DCA curves, the model has better net benefits than a single factor in each cohort.

There are still some limitations in the present study. First, the selection bias from retrospective studies is inevitable. Second, some relevant biomarkers and more detailed treatment information are missing from the SEER database, such as whether TP53 has break-apart translocations or received immunotherapy. The current database does not include surgical information on metastatic lesions, so the model needs to be further validated in a cohort population with better information. Third, the SEER database lacks the records of patients with subsequent LM, causing the incidence of LM in young osteosarcoma patients to be underestimated. Despite these limitations, we believe that this study can help clinicians provide more accurate treatment advice for osteosarcoma patients with LM.

## 5. Conclusion

The present study showed that T stage, N stage, and bone metastasis were independent diagnostic factors of LM for children and young adult osteosarcoma patients. In addition, chemotherapy, primary site, surgery, age, and bone metastasis were associated with prognosis. Two nomograms were established, and considerable predictive performance was obtained.

## Figures and Tables

**Table 1:** Clinical characteristics of osteosarcoma patients.

	Training set (*n* = 434)	Validation set I (*n* = 184)	Validation set II (*n* = 131)	Χ^2^/*Z*	*PP*
Age, years				1.552	0.817
0–8	43 (9.91%)	14 (7.61%)	12 (9.16%)		
9–16	237 (54.61%)	100 (54.35%)	67 (51.15%)		
17–25	154 (35.48%)	70 (38.04%)	52 (39.69%)		

Race				458.173	<0.001
Black	77 (17.74%)	28 (15.22%)	0 (0.00%)		
White	316 (72.81%)	135 (73.37%)	0 (0.00%)		
Other	41 (9.45%)	21 (11.41%)	131 (100.00%)		

Sex				3.028	0.220
Female	190 (43.78%)	85 (46.20%)	48 (36.64%)		
Male	244 (56.22%)	99 (53.80%)	83 (63.36%)		

Primary site				6.525	0.367
Upper limb	59 (13.59%)	28 (15.22%)	19 (14.50%)		
Lower limb	327 (73.35%)	133 (72.28%)	91 (69.47%)		
Other	30 (6.91%)	9 (4.89%)	10 (7.63%)		
Spine/pelvis	18 (4.15%)	14 (7.61%)	11 (8.40%)		

T stage				2.817	0.245
T1	181 (41.71%)	68 (36.96%)	61 (46.56%)		
T2	234 (53.92%)	105 (57.07%)	63 (48.09%)		
T3	10 (2.30%)	6 (3.26%)	3 (2.29%)		
TX	9 (2.07%)	5 (2.72%)	4 (3.05%)		

N stage				0.895	0.639
N0	408 (94.01%)	171 (92.93%)	120 (91.60%)		
N1	13 (3.00%)	9 (4.89%)	8 (6.11%)		
NX	13 (3.00%)	4 (2.17%)	3 (2.29%)		

Tumor size, mm				1.478	0.830
0–100	237 (54.61%)	101 (65.89%)	79 (60.31%)		
101–200	151 (34.79%)	65 (35.33%)	40 (30.53%)		
>200	46 (10.60%)	18 (9.78%)	12 (9.16%)		

Bone metastasis				2.423	0.298
No	418 (96.31%)	176 (95.65%)	122 (93.13%)		
Yes	16 (3.69%)	8 (4.35%)	9 (6.87%)		

Lung metastasis				8.368	0.015
No	343 (79.03%)	161 (87.50%)	99 (80.51%)		
Yes	91 (20.97%)	23 (12.50%)	32 (19.49%)		

Validation set I: internal validation set; validation set II: external validation set.

**Table 2:** Univariate and multivariate logistic analyses of LM in osteosarcoma patients.

	Univariate analysis			Multivariate analysis		
	OR	95% CI	*P*	OR	95% CI	*P*
Age, years						
0–8	Reference					
9–16	1.224	0.554–2.703	0.617			
17–25	0.697	0.297–1.638	0.408			

Race						
Black	Reference					
White	0.849	0.469–1.538	0.589			
Other	0.795	0.312–2.025	0.630			

Sex						
Female	Reference					
Male	1.049	0.658–1.672	0.842			

Primary site						
Lower limb	Reference					
Upper limb	1.252	0.658–2.380	0.494			
Other	0.565	0.191–1.672	0.302			
Spine/pelvis	0.459	0.103–2.044	0.307			

T						
T1	Reference			Reference		
T2	4.166	2.283–7.603	<0.001	3.871	2.052–7.300	<0.001
T3	11.067	2.876–42.583	<0.001	4.739	0.876–25.653	0.071
TX	38.733	7.380–203.290	<0.001	18.915	3.023–118.342	0.002

N						
N0	Reference				Reference	
N1	26.106	5.663–120.346	<0.001	15.855	2.932–85.742	0.001
NX	10.680	3.200–35.645	<0.001	9.123	2.440–34.107	0.001

Tumor size, mm						
0–100	Reference					
101–200	2.005	1.183–3.401	0.010			
＞200	6.406	3.221–12.742	<0.001			

Bone metastasis						
No	Reference			Reference		
Yes	18.889	5.256–67.887	<0.001	13.966	3.423–56.976	<0.001

OR: odds ratio; CI: confidence interval; LM: lung metastasis.

**Table 3:** Clinical characteristics of 114 osteosarcoma patients with LM.

	Training set (*n* = 82)	Validation set I (*n* = 32)	Validation set II (*n* = 32)	Χ^2^/*Z*	*P*
Age, years				5.992	0.200
0–8	4 (4.88%)	6 (18.75%)	4 (12.50%)		
9–16	53 (64.63%)	18 (56.25%)	21 (65.63%)		
17–25	25 (30.49%)	8 (25.00%)	7 (21.88%)		

Race				98.692	<0.001
Black	17 (20.73%)	5 (15.63%)	0 (0.00%)		
White	57 (69.51%)	24 (75.00%)	0 (0.00%)		
Other	8 (9.76%)	3 (9.38%)	32 (100.00%)		

Sex				2.052	0.359
Female	39 (47.56%)	11 (34.38%)	12 (37.50%)		
Male	43 (52.44%)	21 (65.63%)	20 (62.50%)		

Primary site				3.272	0.774
Upper limb	13 (15.85%)	4 (12.50%)	4 (12.50%)		
Lower limb	62 (75.61%)	25 (78.13%)	23 (71.88%)		
Other	2 (2.44%)	2 (6.25%)	3 (9.38%)		
Spine/pelvis	5 (6.10%)	1 (3.13%)	2 (6.25%)		

Grade				1.308	0.520
II	1 (1.22%)	0 (0.00%)	0 (0.00%)		
III	33 (40.24%)	10 (31.25%)	14 (43.75%)		
IV	48 (58.54%)	22 (68.75%)	18 (56.25%)		

Histological type				0.186	0.911
Osteosarcoma	61 (74.39%)	23 (71.88%)	25 (78.13%)		
Chondroblastic osteosarcoma	14 (17.07%)	6 (18.75%)	3 (9.38%)		
Other	5 (6.10%)	1 (3.13%)	1 (3.13%)		
Central osteosarcoma	2 (2.44%)	2 (6.25%)	3 (9.38%)		

T stage				5.985	0.428
T1	13 (15.85%)	4 (12.50%)	8 (25.00%)		
T2	54 (65.85%)	25 (78.13%)	18 (56.25%)		
T3	4 (4.88%)	2 (6.25%)	3 (9.38%)		
TX	11 (13.41%)	1 (3.13%)	3 (9.38%)		

N stage				1.094	0.895
N0	64 (78.05%)	23 (71.88%)	25 (78.13%)		
N1	12 (14.63%)	5 (15.63%)	5 (15.63%)		
NX	6 (7.32%)	4 (12.50%)	2 (6.25%)		

Surgery				0.621	0.733
Yes	67 (81.71%)	28 (87.50%)	26 (81.25%)		
No	15 (18.29%)	4 (12.50%)	6 (18.75%)		

Chemotherapy				6.082	0.048
Yes	78 (95.12%)	30 (93.75%)	26 (81.25%)		
No	4 (4.88%)	2 (6.25%)	6 (18.75%)		

Radiotherapy				2.091	0.351
Yes	9 (10.98%)	2 (6.25%)	1 (3.13%)		
No	73 (89.02%)	30 (93.75%)	31 (96.88%)		

Tumor size, mm				1.219	0.544
0–100	30 (35.59%)	11 (34.38%)	15 (46.88%)		
101–200	31 (37.80%)	14 (43.75%)	11 (34.38%)		
>200	21 (25.61%)	7 (21.88%)	6 (18.75%)		

Bone metastasis				1.974	0.373
No	69 (84.15%)	28 (87.50%)	24 (75.00%)		
Yes	13 (15.85%)	4 (12.50%)	8 (25.00%)		

LM: lung metastasis; validation set I: internal validation set; validation set II: external validation set.

**Table 4:** Univariate and multivariate Cox analyses of osteosarcoma patients with LM.

	Univariate analysis			Multivariate analysis		
	HR	95% CI	*P*	HR	95% CI	*P*
Age, years						
0–8	Reference				Reference	
9–16	0.314	0.109–0.904	0.032	0.264	0.081–0.861	0.027
17–25	0.652	0.221–1.922	0.438	0.621	0.196–1.971	0.419

Race						
Black	Reference					
Other	0.474	0.173–1.300	0.147			
White	0.509	0.281–0.922	0.026			

Sex						
Female	Reference					
Male	1.163	0.688–1.968	0.573			

Primary site						
Lower limb	Reference			Reference		
Other	3.659	0.861–15.555	0.079	6.866	1.538–30.655	0.012
Spine/pelvis	2.858	1.099–7.434	0.031	2.126	0.664–6.810	0.204
Upper limb	2.097	1.070–4.112	0.031	2.138	0.998–4.580	0.051

Grade						
II	Reference					
III	0.264	0.035–2.012	0.198			
IV	0.321	0.043–2.415	0.270			

Histological type						
Osteosarcoma	Reference					
Chondroblastic osteosarcoma	0.823	0.401–1.686	0.594			
Other	1.010	0.312–3.271	0.987			
Central osteosarcoma	<0.001	0–999	0.967			

T						
T1	Reference					
T2	0.877	0.420–1.829	0.726			
T3	1.525	0.411–5.650	0.528			
TX	1.790	0.724–4.427	0.208			

N						
N0	Reference					
N1	2.193	1.120–4.294	0.022			
NX	1.559	0.614–3.956	0.350			

Surgery						
No	Reference				Reference	
Yes	0.330	0.179–0.609	<0.001	0.400	0.188–0.855	0.018
Chemotherapy						
No	Reference				Reference	
Yes	0.097	0.033–0.287	<0.001	0.123	0.037–0.411	0.001

Radiotherapy						
No	Reference					
Yes	1.484	0.666–3.303	0.334			

Tumor size, mm						
0–100	Reference					
101–200	1.398	0.754–2.593	0.287			
>200	1.270	0.644–2.501	0.490			

Bone metastasis						
No	Reference			Reference		
Yes	3.128	1.580–6.192	0.001	3.981	1.695–9.350	0.002

HR: hazard ratio; CI: confidence interval; LM: lung metastasis.

## Data Availability

The datasets generated and/or analyzed during the current study are available in the SEER database (https://seer.cancer.gov/).

## Abbreviations

AUC: Area under the curve
LM: Lung metastasis
SEER: Surveillance, Epidemiology, and End Results.

## Supplementary Materials

Supplementary Materials Supplementary Figure 1: comparison of decision curve analysis between the diagnostic nomogram and the AJCC stage in the training set (a), internal validation set (b), and external validation set (c). Supplementary Figure 2: comparison of decision curve analysis between the prognostic nomogram and the AJCC stage. 12-month, 24-month, and 36-month survival rates in the training set (a-c), internal validation set (d-f), and external validation set (g-i).
