# Supplementary material for: Development and Validation of Nomograms to Assess Risk Factors and Overall Survival Prediction for Lung Metastasis in Young Patients with Osteosarcoma: A SEER-Based Study
Source: Int J Clin Pract. 2022 Oct 25;2022:8568724. doi: 10.1155/2022/8568724 (PMC9626197; doi:10.1155/2022/8568724)
Supplement: Supplementary Materials — Supplementary Figure 1: comparison of decision curve analysis between the diagnostic nomogram and the AJCC stage in the training set (a), internal validation set (b), and external validation set (c). Supplementary Figure 2: comparison of decision curve analysis between the prognostic nomogram and the AJCC stage. 12-month, 24-month, and 36-month survival rates in the training set (a-c), internal validation set (d-f), and external validation set (g-i). [file 8568724.f1.pdf]

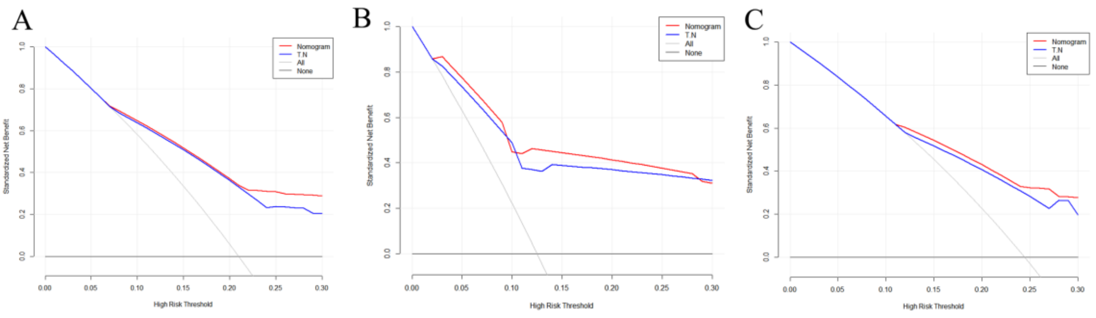

Supplementary Figure 1：Comparison of decision curve analysis between the diagnostic nomogram and AJCC stage in training set (A), internal validation set (B), and external validation set (C).

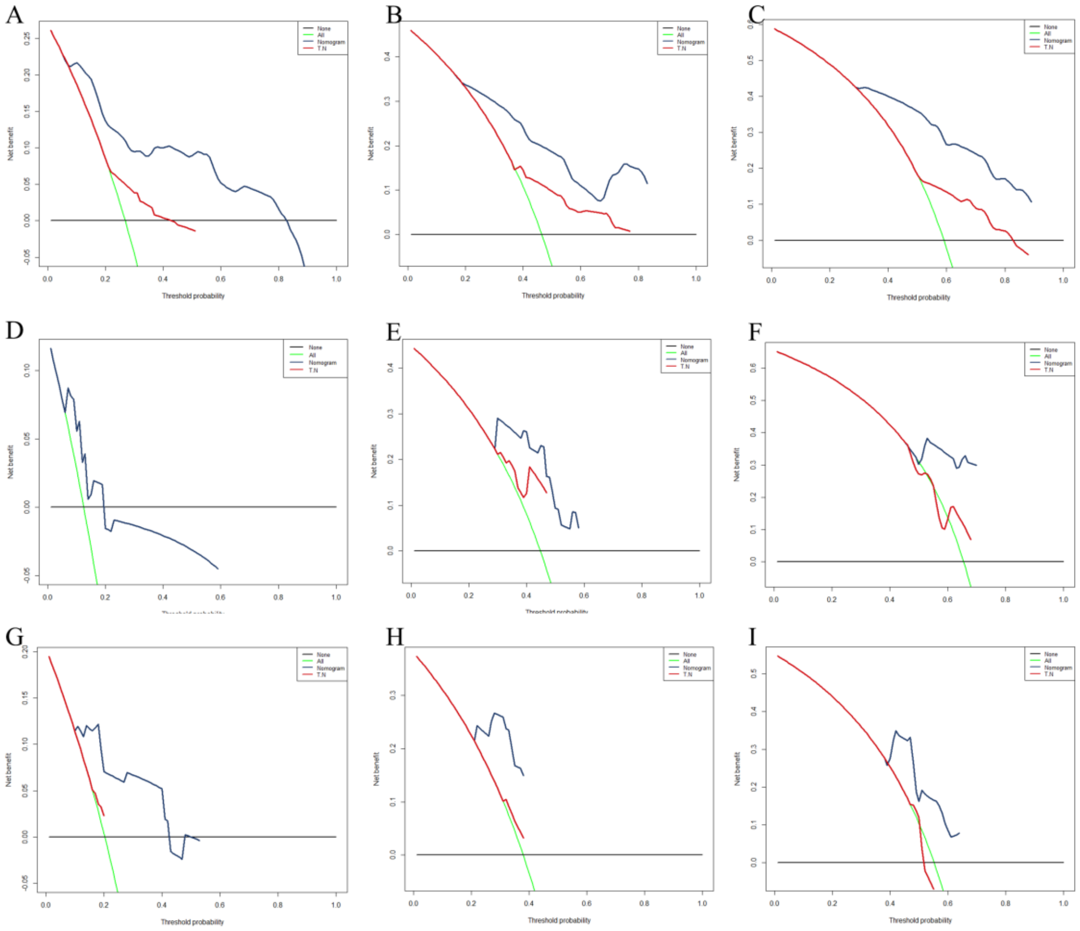

Supplementary Figure 2：Comparison of decision curve analysis between the prognostic nomogram and AJCC stage. 12-, 24-, and 36-month survival in the training set (A-C), internal validation set (D-F), and external validation set (G-I).
